# A Retrospective Study of Postictal Suppression during Electroconvulsive Therapy

**DOI:** 10.3390/jcm11051440

**Published:** 2022-03-05

**Authors:** Virginie Moulier, Julien Guehl, Emilie Evêque-Mourroux, Pierre Quesada, Maud Rothärmel

**Affiliations:** 1 University Department of Psychiatry, Centre d’Excellence Thérapeutique, Institut de Psychiatrie, Centre Hospitalier du Rouvray, 76301 Sotteville-lès-Rouen, France; julien.guehl@ch-lerouvray.fr (J.G.); emilie.eveque-mourroux@ch-lerouvray.fr (E.E.-M.); pierre.quesada@ch-lerouvray.fr (P.Q.); maud.rotharmel@ch-lerouvray.fr (M.R.); 2 Unité de Recherche Clinique, Etablissement Publique de Santé deVille Evrard, 93332 Neuilly-sur-Marne, France

**Keywords:** electroconvulsive therapy (ECT), treatment-resistant depression (TRD), postictal suppression, electroencephalography (EEG)

## Abstract

Background: electroconvulsive therapy (ECT) is the most effective treatment in treatment-resistant depression (TRD), but its response remains partial. Identifying useful indicators to guide decision making for treatment and improve clinical response remains a major issue. The objective of the present retrospective study was to determine if clinical response—early (after 5 ECT sessions) or longer-term (after 12 ECT sessions)—was associated with postictal suppression during the first ECT course and/or with postictal suppression frequency during the whole ECT course. Methods: in a retrospective study, the data of 42 patients suffering from treatment-resistant depression and receiving at least 5 ECT sessions were collected. Two sessions per week of bitemporal brief-pulse ECT sessions were administered to patients. Each of the electroencephalography (EEG) recordings were assessed to determine the presence of postictal suppression. Results: the postictal suppression from the first ECT session predicted a better long-term clinical response (after 12 ECT sessions), but not early clinical response (after only 5 ECT sessions). The postictal suppression frequency was associated with neither the short- nor the long-term clinical response. In addition, postictal suppression and short-term cognitive performances were not associated. Conclusions: this EEG indicator is clinically useful if it appears in the first ECT sessions, but it is no longer relevant in the following sessions.

## 1. Introduction

It is widely accepted that electroconvulsive therapy (ECT) is the most effective treatment in treatment-resistant depression (TRD). However, its response may vary from patient to patient, with response rates ranging from 39% to 85% in patients with previous pharmacotherapy failure [1]. Identifying useful data to guide decision making for treatment techniques and improve clinical responses remains a major issue. Current guidelines recommend electroencephalography (EEG) monitoring of the seizure to assess seizure quality across the course of ECT treatment [2]. According to a recent review of the literature, postictal suppression seems to be the ictal EEG index most frequently associated with better clinical response [3]. Postictal suppression or electrical silence is the period of suppression of bioelectric activity following seizure termination.

This inhibition is due to the release of inhibitory neurotransmitters, with the greatest evidence indicating a role for gamma aminobutyric acid (GABA) [4]. Francis-Taylor et al. (2020) highlighted that the significant relationship between postictal suppression [3] and clinical outcome was mainly demonstrated by multivariate ictal EEG models [5,6,7,8] (i.e., integrating several EEG variables), precluding quantitative examination with a meta-analysis. Nevertheless, several studies have established that clinical improvement is directly associated with greater postictal bioelectric suppression in univariate predictive models. Among these studies, the clinical outcome was measured at variable times: (i) at the very start of the ECT course, after two [9] or three ECT sessions [10]; (ii) during the ECT course, after six ECT sessions [11,12]; (iii) and after the end of the ECT course [13,14,15]. In addition, postictal suppression was studied either during one specific ECT session—usually the first or second ECT session [7,9,10,12], allowing researchers to test whether this index was a predictor of response to ECT—or throughout the different sessions, exploring relationships between postictal suppression and clinical outcome [5,6,8,11,13,14,15]. This methodological variability does not allow researchers to clearly establish whether postictal suppression predicts the clinical response in the short, medium or long term.

Until now, few studies have focused on relationships between ictal EEG measures and the side-effects of ECT. To our knowledge, only Perera et al. (2004) included cognitive outcome measures to assess whether peri-ictal EEG features were associated with the cognitive effects of ECT [14]. No significant association was demonstrated between EEG features and acute (immediately after each ECT session) or short-term (2–7 days following the course of ECT) cognitive effects.

With these issues in mind, the aim of the present retrospective study is to determine if clinical response—early (after 5 ECT) or longer-term (after 12 ECT)—is associated with postictal suppression during the first ECT, or with postictal suppression frequency during the whole ECT course. In the second aim, the evolution of postictal suppression over the ECT sessions is studied. In the third and final aim, the relationship between cognition side effects and postictal suppression is explored. We hypothesize that postictal suppression during the first ECT predicts an early and longer-term clinical response, and that postictal suppression frequency during the whole ECT course is significantly associated with early and longer-term clinical response.

## 2. Methods

### 2.1. Participants

In this retrospective study, information was extracted from clinical files of Rouvray Hospital in Sotteville-Lès-Rouen (France). Participants were inpatients referred for ECT by their psychiatrist for treatment-resistant depression. These patients typically failed to achieve a clinical response to three separate trials of antidepressants from different classes (at least one of which was a tricyclic) at sufficient dose for at least six weeks, according to stage III of Thase and Rush criteria [16]. The number of ECT sessions to be administered was based on the patient’s clinical response.

Inclusion criteria for the present study were patients who: (i) were aged 18 to 70 years; (ii) had a current DSM-IV diagnosis of major depressive episode, with a 21-item Hamilton Depression Rating Scale (HDRS) score of at least 15 [17]; (iii) had received at least 5 ECT sessions; and (iv) had sufficient knowledge of the French language for the clinical and cognitive assessments. Patients were excluded from the study if: (i) they had already received ECT treatment for the current episode; (ii) their pharmacological treatment (antidepressants and/or mood stabilizers) were not stable during ECT treatment; and (iii) if they were suffering from a neurological disorder.

Informed consent was obtained from all patients involved in the study. Under French ethical law (public health code), retrospective studies based on the exploitation of routine care data do not have to be submitted to an ethics committee. This study was conducted in accordance with the declaration of Helsinki.

### 2.2. Electroconvulsive Therapy

ECT treatment was delivered using a MECTA Spectrum 5000 Q (MECTA Corp, Tualatin, OR, USA) or a Thymatron System IV device (Somatics Inc., Lake Bluff, IL, USA) and used bitemporal brief-pulse ECT sessions with two sessions per week. The seizure threshold was determined by an individual titration method during the first ECT session [18]. A maximum of three stimulations was allowed during the titration sequence. The initial stimulation was administered at twice the seizure threshold, then was permitted to be increased during the course of treatment, if the session was considered ineffective. In such cases, another stimulation with a one-step increase of 50% was delivered. A session was considered effective if the EEG seizure lasted either longer than 20 s or 15 to 20 s followed by a postictal suppression [13]. The anesthetic was propofol (2,6-di-isopropylphenol) at doses of 1 to 2 mg/kg, with curare (suxamethonium chloride) at doses of 0.3 to 0.8 mg/kg for short-term paralysis.

### 2.3. Postictal Suppression Determination

Each EEG was assessed by two psychiatrists and one senior psychiatrist with over 10 years of experience in the field of ECT. In case of disagreements between the two psychiatrists, the senior psychiatrist made the decision. Postictal suppression was defined as a flat line (at least 5 s) in the period immediately following the electrically-induced seizure. The postictal suppression frequency was calculated by dividing the number of sessions where the patient had postictal suppression by the total number of sessions administered to the patient.

### 2.4. Assessments

HDRS was used to assess depressive symptoms before ECT, and after 5 and 12 ECT sessions [17]. Assessing after 5 ECT sessions seems to be a good indicator of early response to ECT; this is because the decrease in depression scores over the ECT treatment course was steep and regular during the first five ECT sessions, before becoming more flattened [19]. The relative improvement was calculated using the following index: (Pre-treatment HDRS score minus Post-treatment HDRS score)/Pre-treatment HDRS score. All patients had a cognitive assessment before and during ECT treatment (within 24 h after the fifth ECT session) with the following tests: (i) the Mini-Mental State Examination to assess global cognitive functioning [20]; (ii) the RL/RI-16 [21] and the Doors test [22] assessing verbal and visual memory performances, respectively; (iii) the D2 test of attention [23]; (iv) and the Rey–Osterrieth complex figure assessing visuospatial/constructional ability, planning, and organization [24].

A Squire Subjective Memory Questionnaire (SSMQ) and Cognitive Failures Questionnaire (CFQ) were self-administered to assess subjective cognitive functioning [25,26].

### 2.5. Statistical Analysis

The relative improvement in HDRS scores—between Pre-treatment and after 5 ECT sessions, and between Pre-treatment and after 12 ECT sessions—were compared between patients who underwent postictal suppression during the first ECT and those who did not, using Mann–Whitney’s nonparametric test; this was because of the small sample size. Size effects were estimated with eta squared (ɳ^2^) [27]. Linear regression was used to explore whether postictal suppression frequency (number of sessions where the patient had postictal suppression ÷ number of sessions administered to the patient) could be associated with clinical improvement after 5 and 12 ECT sessions. Pearson correlation evaluated whether there was any statistical relationship between postictal suppression frequency over the first 5 sessions and the evolution of cognitive performance (between baseline and the 5th ECT session). Bonferroni correction for multiple comparisons was applied due to the high number of tests on cognitive variables. The change in the percentage of patients with postictal suppression over the course of the ECT sessions was tested with Cochran’s Q test. A statistical univariate model was favored, in order to easily conclude whether or not postictal suppression is clinically useful and facilitate future meta-analyses. The analyses were conducted using SPSS, version 28 (IBM, Armonk, NY, USA).

## 3. Results

### 3.1. Sample Characteristics

The data of 42 patients receiving at least 5 ECT sessions were collected. Additional information for up to 12 ECT sessions could be collected among 29 patients. The demographic and clinical characteristics are reported in Table 1. The group of patients for which clinical and EEG data were collected for up to 12 ECT sessions (*n* = 29) did not statistically differ from other patients (*n* = 13) for all characteristics, except for the total number of ECT sessions (mean (SD) = 18.8 (3.7) in the group receiving at least 12 ECT sessions versus 13.5 (5.5) in the other patients; *p* = 0.004). The determination of postictal suppression during the first ECT session was not possible in two patients due to the too-early interruption of the EEG, and the HDRS score after 5 ECT sessions was missing for one patient.

### 3.2. ECT Parameters

The mean (SD) electrical charges in milliCoulombs were: 80.19 (35.32) during the first ECT session (*n* = 42), 263.60 (177.57) during the 5th ECT session (*n* = 42) and 404.34 (331.01) during the 12th ECT session (*n* = 25). According to ECT devices, pulse width varied between 0.3 and 0.5 ms (mean (SD) = 0.38 (0.10)) during the first ECT session and between 0.3 and 1 ms (mean (SD) = 0.44 (0.20)) during the 12th ECT session. The seizure durations in seconds were: 31.62 (26.25) during the first ECT session (*n* = 37), 27.83 (14.43) during the 5th ECT session (*n* = 40) and 29.73 (12.71) during the 12th ECT session (*n* = 30). During the first five ECT sessions (*n* = 42), the mean (SD) percentage of sessions requiring restimulation was 20.95 (22.50).

### 3.3. Relationships between Postictal Suppression during the First ECT Session and Clinical Response

Patients experiencing postictal suppression during the first ECT session, i.e., the session where the seizure threshold was determined by an individual titration method, showed a higher clinical response (mean (SD) = 74.25% (08.18), *n* = 6) after 12 ECT sessions than patients without postictal suppression during the first ECT session (mean (SD) = 55.37% (19.19), *n* = 22; *p* = 0.02, ɳ^2^ = 0.188). For verification purposes, the clinical response after 12 ECT sessions was not different between patients who had seizures during the first ECT session and patients who did not (*p* = 0.835, ɳ^2^ = 0.0019). The clinical response after only 5 ECT sessions was not significantly different between patients experiencing postictal suppression during the first ECT session (mean (SD) = 33.94% (17.11), *n* = 10) and those who did not (mean (SD) = 31.45 (23.31), *n* = 29; *p* = 0.76, ɳ^2^ = 0.0024). Patients with and without postictal suppression during the first ECT session did not significantly differ in their demographic and clinical characteristics (Table 2).

### 3.4. Relationships between Postictal Suppression at Each ECT Session and Clinical Response

The mean (SD) postictal suppression frequency during treatment was 49.29% (29.44) during the first five sessions and 50.86% (21.29) during the 12 sessions. In the linear regression model, postictal suppression frequency during the first five sessions was not significantly associated with the clinical response after 5 ECT sessions (R square = 0.014; B = 0.091; *p* = 0.458). Postictal suppression frequency during the 12 sessions was not associated with the clinical response after 12 sessions ECT either (R square < 0.001; B = 0.003; *p* = 0.989).

### 3.5. Postictal Suppression throughout Treatment

**Figure 1:**
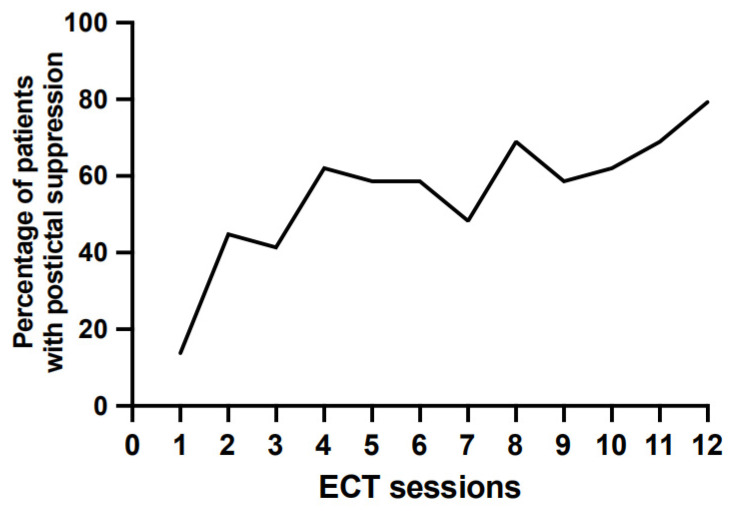
Evolution of the percentage of patients experiencing postictal suppression throughout ECT sessions. Note: the percentages were calculated at each session in the sample of 29 patients treated for at least 12 ECT sessions.

The percentage of patients experiencing postictal suppression significantly increased over the ECT sessions (Q Cochran = 44.66; *p* < 0.001; df = 11; *n* = 29). Only 13.8% of the patients had postictal suppression from the first ECT session, while there were 79.3% in the 12th session (Figure 1). The analysis remained significant if the first session, which involved dosage titration, was excluded (Q Cochran = 18.86; *p* = 0.042).

### 3.6. Relationships between Postictal Suppression and Cognitive Side Effects

The postictal suppression frequency during the first five sessions was not correlated with the evolution of scores of any cognitive tests assessing attention, memory and visuospatial abilities between baseline and after 5 ECT sessions (Table 3). The postictal suppression frequency during the first five sessions was correlated with neither the SSMQ score (r = 0.118; *p* = 0.464; *n* = 41), nor the CFQ score (r = −0.081; *p* = 0.613; *n* = 41).

## 4. Discussion

According to our retrospective study, experiencing postictal suppression from the first ECT session might predict a better long-term clinical response (after 12 ECT sessions). This outcome is consistent with previous studies, which showed that postictal suppression from the first ECT sessions (the first or second ECT session, according to the studies) was associated with a better clinical response at the end of treatment [7,9]. It is, therefore, possible that postictal suppression, rather, predicts the long-term response. However, Gangadhar et al. (1999) highlighted that postictal suppression during the first ECT session could predict remission after only 6 ECT sessions [12].

Regarding postictal suppression frequency (the number of sessions where the patient had postictal suppression/total number of sessions administered to the patient), the absence of a correlation between this index and cognition, especially cognitive impairment, confirms the study of Perera et al. (2004), which found no significant association between peri-ictal EEG features and the acute or short-term cognitive effects of ECT [14]. In addition, the postictal suppression frequency was neither associated with the short- nor the long-term clinical response.

In view of our results, postictal suppression may be considered an indicator of good clinical response, if it appears immediately during the first ECT sessions. However, as the proportion of patients experiencing postictal suppression increased over the course of the sessions, reaching 79.3% of the patients at the 12th ECT session, postictal suppression no longer makes it possible to discriminate between the good (or bad) responders during the following sessions. Postictal suppression accumulation does not seem to be associated with clinical response. As suggested by Perera et al. (2004), the presence of postictal suppression in the first ECT sessions may reflect individual differences in the strength of inhibitory processes that terminate the seizure [14]. Patients with more intense inhibitory processes after a seizure may have greater potential of benefiting from ECT. This higher therapeutic efficacy may be related to genetic differences in the GABA response [28]. As a consequence of the release of GABA, dysfunctional circuits underlying major depression would be reset by an anticonvulsant mechanism [28,29]. Then, the increase in the number of patients experiencing postictal suppression over the course of treatment might indicate an adaptive brain mechanism. This effect might be mediated by GABA release allowing the seizure to terminate. Accumulating sessions might promote GABA release, even in patients with a weak initial response to GABA. Brain levels of GABA might be raised after each ECT session, resulting in increased background inhibitory neurotransmission, thus facilitating the occurrence of postictal suppression in the following sessions. It might induce a reset of dysfunctional circuits, stimulating neuroplasticity and neurogenesis [28]. However, the mechanisms of action of ECT are probably more complex, involving the glutamatergic system and/or other neurotransmitters. This study has some noteworthy limitations. First, this study is retrospective and based on clinical files. It would be interesting to explore this question in a prospective study. The second limitation is that only bitemporal electrode placement was tested. Given the cognitive advantage in a right unilateral ECT placement, this placement should be also investigated [30]. Thirdly, the interpretation of the EEG was made by clinicians, and was not automated, which does not allow us to rule out errors in scoring. Fourth, the cognitive assessment occurred only after 5 ECT sessions, which did not allow evaluation of whether the postictal suppression was associated with long term cognitive side effects. In addition, retrograde amnesia for autobiographical information, which is one of the most critical side effects of ECT, was not assessed in the study. Finally, the group receiving 12 ECT sessions was a subgroup of the whole sample and included only 29 patients due to missing data in the files. In addition, only six patients experiencing postictal suppression during the first ECT session showed a higher clinical response after 12 ECT sessions. We cannot rule out the possibility of a false positive result favored by the small sample size, which relativizes the relevance of these results.

In summary, this retrospective study showed that postictal suppression from the first ECT session might predict a better long-term clinical response (after 12 ECT sessions), but that postictal suppression frequency was neither associated with the short- or long-term clinical response. In addition, postictal suppression and short-term cognitive performance were not associated. This indicator is clinically useful if it appears in the first ECT sessions, but is no longer relevant in the following sessions. However, we currently do not know how to favor the presence of postictal suppression during the first sessions. One possibility would be to administer another neuromodulation technique before ECT sessions, for example, rTMS or transcranial direct current stimulation (tDCS), which might strengthen the GABA system, possibly facilitating the presence of postictal suppression, thus increasing the clinical response. This hypothesis should be investigated in a future study.

## Figures and Tables

**Table 1:** Characteristics of the sample.

	Patients Treated for at Least 5 ECT Sessions	Patients Treated for at Least 12 ECT Sessions
Sample Size	42 patients	29 patients
Age (years): mean (SD)	47.64 (14.64)	47.86 (13.77)
Female sex	59.5%	55.2%
Graduate studies	57.1%	65.5%
Married	50.0%	48.3%
Employed	40.5%	44.8%
Duration of current episode (months)	26.69 (27.18)	23.94 (25.36)
Duration of mood disorder (years)	17.10 (12.15)	15.93 (10.82)
Bipolar disorder	35.7%	37.9%
Suicide Risk	88.1%	86.2%
Baseline HDRS	26.14 (5.43)	25.86 (5.67)
HDRS after 5 ECT sessions	17.49 (7.37)	18.10 (7.54)
HDRS after 12 ECT sessions	-	10.26 (5.17)
Total number of ECT sessions during treatment: mean (SD), range	17.12 (4.92), [5;27]	18.97 (2.13); [13;20]

Notes. Frequencies or means (standard deviation) are reported in the table. The two samples are not independent. The patients of the group receiving 12 ECT sessions also belong to the group receiving 5 ECT sessions. ECT: electroconvulsive therapy; HDRS: Hamilton rating scale for depression.

**Table 2:** Characteristics of the patients with and without postictal suppression during the first ECT session.

	Patients with Postictal Suppression (*n* = 10)	Patients without Postictal Suppression (*n* = 30)	*p* Value
Age (years)	51.90 (17.49)	47.23 (13.16)	0.246 ^1^
Female sex	40.0%	66.7%	0.159 ^2^
Graduate studies	70.0%	53.3%	0.471 ^2^
Married	60.0%	50.0%	0.721 ^2^
Employed	20.0%	46.7%	0.263 ^2^
Duration of current episode (months)	34.14 (34.41)	25.45 (25.15)	0.432 ^1^
Duration of mood disorder (years)	18.50 (14.55)	16.87 (11.56)	0.866 ^1^
Bipolar disorder	40.0%	30.0%	0.700 ^2^
Suicide Risk	80.0%	90.0%	0.584 ^2^
Baseline HDRS	26.40 (6.74)	25.73 (4.96)	0.548 ^1^
Total number of ECT sessions during treatment	16.70 (6.21)	17.40 (4.47)	0.678 ^1^

Notes. Frequencies or means (standard deviation) are reported in the table. ^1^ *p* values are results of Mann–Whitney tests; ^2^ *p* values are results of Fisher′s exact tests. ECT: electroconvulsive therapy.

**Table 3:** Correlation between cognitive performance evolution (after 5 ECT sessions *minus* baseline scores) and the postictal suppression frequency during the 5 ECT sessions.

	Baseline	After 5 ECT Sessions	r	*p*
**MMSE (*n* = 41)**	26.45 (3.04)	26.63 (2.73)	−0.186	0.245
**Doors test (*n* = 38)**				
part A, scaled score	6.51 (3.84)	6.95 (3.97)	−0.322	0.049 ^#^
part B, scaled score	6.90 (3.29)	6.90 (4.16)	−0.286	0.082
**D2 test of attention (*n* = 38)**				
TN: Total number of characters processed	84.80 (8.56)	88.34 (9.65)	0.054	0.749
CP: Concentration performance	91.54 (6.34)	95.74 (6.67)	0.094	0.586
**Rey figure (Z score) (*n* = 37)**	−1.32 (1.98)	−0.97 (2.02)	−0.128	0.451
**RL/RI−16 test (*n* = 38**)				
Free Recall 1 (z score)	−0.79 (0.98)	−0.95 (0.98)	0.083	0.620
Free Recall 2 (z score)	−0.79 (0.90)	−1.19 (1.08)	0.150	0.369
Free Recall 3 (z score)	−0.88 (1.05)	−1.27 (1.28)	0.135	0.420
Delayed Free Recall (z score)	−0.90 (1.05)	−1.98 (1.27)	0.011	0.946

Notes: Means (standard deviations) are reported. The number of subjects included in the analysis is reported for each cognitive variable. ^#^ The result was no longer significant after Bonferroni correction for multiple comparisons. MMSE: Mini-Mental State Examination; Rey figure: Rey–Osterrieth complex figure; ECT: electroconvulsive therapy.

## Data Availability

The data presented in this study are available on request from the corresponding author.

## References

[1] Haq A.U., Sitzmann A.F., Goldman M.L., Maixner D.F., Mickey B.J. (2015). Response of Depression to Electroconvulsive Therapy: A Meta-Analysis of Clinical Predictors. J. Clin. Psychiatry.

[2] Weiss A., Hussain S., Ng B., Sarma S., Tiller J., Waite S., Loo C. (2019). Royal Australian and New Zealand College of Psychiatrists Professional Practice Guidelines for the Administration of Electroconvulsive Therapy. Aust. N. Z. J. Psychiatry.

[3] Francis-Taylor R., Ophel G., Martin D., Loo C. (2020). The Ictal EEG in ECT: A Systematic Review of the Relationships between Ictal Features, ECT Technique, Seizure Threshold and Outcomes. Brain Stimul..

[4] Sackeim H.A. (2004). Convulsant and Anticonvulsant Properties of Electroconvulsive Therapy: Towards a Focal Form of Brain Stimulation. Clin. Neurosci. Res..

[5] Krystal A.D., Weiner R.D., Coffey C.E. (1995). The Ictal EEG as a Marker of Adequate Stimulus Intensity with Unilateral ECT. J. Neuropsychiatry Clin. Neurosci..

[6] Krystal A.D., Coffey C.E., Weiner R.D., Holsinger T. (1998). Changes in Seizure Threshold Over the Course of Electroconvulsive Therapy Affect Therapeutic Response and Are Detected by Ictal EEG Ratings. JNP.

[7] Kimball J.N., Rosenquist P.B., Dunn A., McCall V. (2009). Prediction of Antidepressant Response in Both 2.25×threshold RUL and Fixed High Dose RUL ECT. J. Affect. Disord..

[8] Azuma H., Yamada A., Shinagawa Y., Nakano Y., Watanabe N., Akechi T., Furukawa T.A. (2011). Ictal Physiological Characteristics of Remitters during Bilateral Electroconvulsive Therapy. Psychiatry Res..

[9] Nobler M.S., Luber B., Moeller J.R., Katzman G.P., Prudic J., Devanand D.P., Dichter G.S., Sackeim H.A. (2000). Quantitative EEG During Seizures Induced by Electroconvulsive Therapy: Relations to Treatment Modality and Clinical Features. I. Global Analyses. J. ECT.

[10] Jagadisha B.N., Gangadhar B., Janakiramiah N., Girish K., Ramakrishnan A. (2003). Post-Seizure EEG Fractal Dimension and Spectral Power Predict Antidepressant Response to Unilateral ECT. Indian J. Psychiatry.

[11] Suppes T., Webb A., Carmody T., Gordon E., Gutierrez-Esteinou R., Hudson J.I., Pope H.G. (1996). Is Postictal Electrical Silence a Predictor of Response to Electroconvulsive Therapy?. J. Affect. Disord..

[12] Gangadhar B.N., Subbakrishna D.K., Janakiramaiah N., Motreja S., Dutt D.N., Paramehwara G. (1999). Post-Seizure EEG Fractal Dimension of First ECT Predicts Antidepressant Response at Two Weeks. J. Affect. Disord..

[13] Nobler M.S., Sackeim H.A., Solomou M., Luber B., Devanand D.P., Prudic J. (1993). EEG Manifestations during ECT: Effects of Electrode Placement and Stimulus Intensity. Biol. Psychiatry.

[14] Perera T.D., Luber B., Nobler M.S., Prudic J., Anderson C., Sackeim H.A. (2004). Seizure Expression During Electroconvulsive Therapy: Relationships with Clinical Outcome and Cognitive Side Effects. Neuropsychopharmacology.

[15] Azuma H., Fujita A., Sato K., Arahata K., Otsuki K., Hori M., Mochida Y., Uchida M., Yamada T., Akechi T. (2007). Postictal Suppression Correlates with Therapeutic Efficacy for Depression in Bilateral Sine and Pulse Wave Electroconvulsive Therapy. Psychiatry Clin. Neurosci..

[16] Thase M.E., Rush A.J. (1997). When at First You Don’t Succeed: Sequential Strategies for Antidepressant Nonresponders. J. Clin. Psychiatry.

[17] Hamilton M. (1980). Rating Depressive Patients. J. Clin. Psychiatry.

[18] Sackeim H.A., Long J., Luber B., Moeller J.R., Prohovnik I., Devanand D.P., Nobler M.S. (1994). Physical Properties and Quantification of the ECT Stimulus: I. Basic Principles. Convuls. Ther..

[19] Kellner C.H., Knapp R., Husain M.M., Rasmussen K., Sampson S., Cullum M., McClintock S.M., Tobias K.G., Martino C., Mueller M. (2010). Bifrontal, bitemporal and right unilateral electrode placement in ECT: Randomized trial. Br. J. Psychiatry J. Ment. Sci..

[20] Folstein M.F., Folstein S.E., McHugh P.R. (1975). Mini-Mental State. J. Psychiatr. Res..

[21] Grober E., Buschke H., Crystal H., Bang S., Dresner R. (1988). Screening for Dementia by Memory Testing. Neurology.

[22] Baddeley A.D., Wilson B.A., Kopelman M.D. (1995). Handbook of Memory Disorders.

[23] Brickenkamp R. (1996). Test D2. Test D’attention Concentrée.

[24] Osterrieth P.A. (1944). Le Test de Copie d’une Figure Complexe: Contribution à l’étude de La Perception et de La Mémoire. Arch. Psychol..

[25] Squire L.R., Wetzel C.D., Slater P.C. (1979). Memory Complaint after Electroconvulsive Therapy: Assessment with a New Self-Rating Instrument. Biol. Psychiatry.

[26] Broadbent D.E., Cooper P.F., FitzGerald P., Parkes K.R. (1982). The Cognitive Failures Questionnaire (CFQ) and Its Correlates. Br. J. Clin. Psychol..

[27] Fritz C.O., Morris P.E., Richler J.J. (2012). Effect Size Estimates: Current Use, Calculations, and Interpretation. J. Exp. Psychol. Gen..

[28] Seymour J. (2021). Commentary and Update on the Contribution of the GABA Hypothesis to Understanding the Mechanism of Action of Electroconvulsive Therapy. J. ECT.

[29] Farzan F., Boutros N.N., Blumberger D.M., Daskalakis Z.J. (2014). What does the electroencephalogram tell us about the mechanisms of action of ECT in major depressive disorders?. J. ECT.

[30] Su L., Jia Y., Liang S., Shi S., Mellor D., Xu Y. (2019). Multicenter Randomized Controlled Trial of Bifrontal, Bitemporal, and Right Unilateral Electroconvulsive Therapy in Major Depressive Disorder. Psychiatry Clin. Neurosci..

